# Longitudinal Predictors of Functional Impairment in Older Adults in Europe – Evidence from the Survey of Health, Ageing and Retirement in Europe

**DOI:** 10.1371/journal.pone.0146967

**Published:** 2016-01-19

**Authors:** André Hajek, Hans-Helmut König

**Affiliations:** Department of Health Economics and Health Services Research, Hamburg Center for Health Economics, University Medical Center Hamburg-Eppendorf, Hamburg, Germany; Federal University of Rio de Janeiro, BRAZIL

## Abstract

**Objective:**

To examine time-dependent predictors of functional impairment in older adults in Europe longitudinally.

**Methods:**

Data were derived from the Survey of Health Ageing, and Retirement in Europe (2004–2013). Functional impairment was assessed by using activities of daily living (ADL) and instrumental activities of daily living (IADL) indices. Fixed effects regressions were used to estimate the effects of sociodemographic factors (age, marital status, living situation, and income deciles (median split)), lifestyle factors (smoking status and alcohol consumption per week), depression, cognitive function and chronic diseases on the outcome variables.

**Results:**

Longitudinal regressions revealed that functional impairment increased significantly with age, the occurrence of depression, cognitive impairment, the number of chronic conditions, and less than daily alcohol consumption in the total sample and in both sexes. Moreover, the onset of smoking and living without a spouse/partner in household increased functional impairment in the total sample. The effect of depression on functional impairment was significantly more pronounced in men.

**Conclusion:**

Our findings highlight the relevance of changes in age, depression, cognitive function, smoking and chronic diseases for functional impairment. Since particularly depression and smoking may be avoidable, developing strategies to prevent depression or stop smoking might be useful approaches to postpone functional impairment in older adults.

## Introduction

Functional impairments (FI) refer to deteriorations in managing basal (e.g. bathing, dressing or using the toilet) as well as instrumental activities of daily living (e.g. using a telephone or taking medication). Functional decline is associated with numerous adverse health outcomes, such as institutionalization [1, 2] or mortality [3, 4].

It is projected that the proportion of older adults will markedly increase in Europe in the next decades. Due to these demographic changes, it is most likely that the number of individuals with FI will increase considerably in the same period. Consequently, interventional strategies are urgently needed.

Most of the previous studies examining predictors of FI were based on cross-sectional data [5–7]. Only a few longitudinal studies were conducted thus far [8–11]. However, most of these longitudinal studies used a *static* set of baseline characteristics as predictors. Therefore, it is almost unknown how *changes* [12–14] in independent variables affect functional decline.

Thus, we aimed at determining how various factors affect FI in older adults in Europe. To this end, we analyzed time-dependent variables which are supposed to be important for functional decline, including sociodemographic factors [15, 16], lifestyle factors [17–19], cognitive [11, 20] as well as mental factors [9] and comorbidity [6].

Based on these studies, we hypothesize that increasing chronic conditions (e.g. osteoarthritis, hip fracture, Parkinson disease) increase functional decline. The onset of these chronic conditions might lead to the inability to perform activities of daily living.

Moreover, we hypothesize that the onset of cognitive decline and the occurrence of depression increase functional decline. For example, the occurrence of cognitive decline might lead to the inability to use the telephone. Furthermore, the hypothesis that the onset of depression lead to functional decline can be explained by decreased physical activity and social interaction [21, 22]. As for lifestyle factors, we hypothesize that a bad lifestyle in terms of alcohol consumption and smoking lead to functional decline. Moreover, we hypothesize that age has an independent effect on functional decline [23].

By using fixed effects (FE) regressions (a panel data method), the potential of longitudinal data can be exploited and thus insights into the causal relationship can be derived. Furthermore, unobserved time-constant factors can be taken into account (in order to avoid omitted variable bias), providing consistent estimates (under the assumption of strict exogeneity). In a second step, this might open up possibilities for prevention or postponement of the onset of functional decline.

## Methods

### Sample

Data were derived from wave 1 (2004–2005), 2 (2006–2007), 4 (mainly in 2011) and 5 (2013) of the Survey of Health Ageing, and Retirement in Europe (SHARE) [24]. The third wave (2008–2009), also known as SHARELIFE, was excluded from analysis as it concentrated on the life histories of the participants (retrospective).

Based on probability samples, non-institutionalized individuals aged 50 years and above as well as their spouses (regardless of age) were interviewed in 12 (wave 1), 15 (wave 2 and wave 5) or 16 (wave 4) European countries, in total: Austria, Germany, Sweden, Netherlands, Spain, Italy, France, Denmark, Greece, Switzerland, Belgium, Israel, Czechia, Poland, Ireland, Luxembourg, Hungary, Portugal, Slovenia and Estonia. The sample is representative of community-dwelling individuals in old age. Data were collected by computer-assisted personal interviews, except for drop off and vignettes questionnaires, which were conducted via paper and pencil. Please see Börsch-Supan et al. for further details [25].

During waves 1 to 4, SHARE has been reviewed and approved by the Ethics Committee of the University of Mannheim several times and most recently in 2010. Wave 4 of SHARE and the continuation of the project in wave 5 have been reviewed and approved by the Ethics Council of the Max-Planck-Society for the Advancement of Science (most recently in 2012). Prior to the CAPI interview consent was given verbal and then documented by the interviewers. This consent procedure was approved by the ethics committees. The ethical commission agreed that a verbal consent is sufficient and that written consent statements are not necessary for the conduction of SHARE interviews. Please note that in some of the SHARE countries (depending on country-specific legislation) respondents had to sign a written consent statement for re-contact. Additionally, the respondents´ written consent was necessary for the collection of Dried Blood Spots and for the linkage of the SHARE survey data to administrative data.

### Functional impairment

Two self-reported measures of impairments in basic activities and two measures of impairments in instrumental activities of daily living were used (each with “any difficulty”: 0 = no, 1 = yes). The first score (“Activities of Daily Living Wallace and Herzog Index” [26], ADL I) is the sum of the tasks “dressing”, “bathing/showering” and “eating, cutting up food”, ranging from 0 to 3. The second score (“Activities of Daily Living Index“, ADL II) is the sum of the tasks “dressing“, “bathing/showering”, “eating, cutting up food”, “walking across a room” and “getting in or out of bed” with values from 0 to 5. The scales were adapted from Katz et al. [27].

The third score (“Instrumental Activities of Daily Living Index”, IADL I) is the sum of “telephone calls”, “taking medications” and “managing money”. Moreover, the fourth score (“Instrumental Activities of Daily Living Index 2”, IADL II) is the sum of “telephone calls”, “taking medications”, “managing money”, “shopping for groceries” and “preparing a hot meal” with values from 0 to 5. The scales were adapted from Lawton and Brody [28].

The scores were the sum of activities individuals reported difficulties in performing. This means, the higher the score, the higher the impairments. It is worth noting that the scales were validated [26, 29]. Moreover, they were used to increase comparability with US-measures and the RAND Health and Retirement Study (HRS) [30].

### Independent variables

Sex, age, marital status (married and living together with spouse; registered partnership; married, living separated from spouse; never married; divorced; widowed), living situation (Ref.: living with a spouse/partner in household; living without spouse/partner in household) and income (household income percentiles; dichotomized by median split) were used as independent variables. Marital status was dichotomized in regression analysis (0 = married and living together with spouse / registered partnership; 1 = married, living separated from spouse; never married; divorced; widowed). Furthermore, lifestyle factors (current smoking status (yes; no) and alcohol consumption) were included. Alcohol consumption was quantified as alcohol days a week in the last three months (“not at all” to “almost every day”). It was dichotomized (daily alcohol consumption (“almost every day”) vs. less than daily alcohol consumption (otherwise)).

In addition, cognitive function was quantified by using the item: “Now please tell me all the words you can recall (up to one minute for recall with ten words list: 1. Butter, 2. Arm, 3. Letter, 4. Queen, 5. Ticket, 6. Grass, 7. Corner, 8. Stone, 9. Book, 10. Stick)” (adapted from the Ten-Word Delay Recall Test [31]), with a possible range from 0 (worst) to 10 (best). Depression was quantified by using the European Depression (EURO-D) 12-item scale (e.g. “In the last month, have you cried at all?”, with no = 0, yes = 1), ranging from 0 to 12 with a higher score indicating more depressive symptoms. The scale was developed and validated to compare depressive symptoms across different European centers in order to account for differences between regions. The EURO-D scale was dichotomized (1 if EURO-D ≥ 4; 0 otherwise [32]). Thereby, we focus on transitions to depression. Moreover, comorbidity was quantified by the number of chronic diseases (heart attack; high blood pressure or hypertension; high blood cholesterol; stroke or cerebral vascular disease; diabetes or high blood sugar; chronic lung disease; arthritis, including osteoarthritis, or rheumatism; cancer or malignant tumor; stomach or duodenal ulcer, peptic ulcer; Parkinson’s disease; cataracts; hip fracture or femoral fracture).

Additionally, for descriptive purposes, the level of education (a time-constant variable) was assessed by using the International Standard Classification of Education [33] (ISCED-97, with six levels from “primary education or first stage of basic education” to “second stage of tertiary education”). It is worth noting that time-constant variables cannot be included as independent variables in FE regression models. Moreover, we did not control for country of origin since it is also a time-constant variable.

### Statistical analysis

By using panel econometric regressions, time-constant unobserved heterogeneity (e.g. personality) can be taken into account. This is a major advantage over cross-sectional regressions. As indicated by Sargan-Hansen test (a Hausman test with cluster-robust standard errors), random effects regressions are inconsistent [34]. This can be explained by unobserved time-constant factors which are correlated with the predictors [35]. Consequently, (linear) fixed effects (FE) regression are the model of choice because they provide consistent estimates if this correlation is present [34]. FE regressions (also called “Within”-regressions) solely use intraindividual changes over time (for technical details: [36]). It is worth mentioning that solely time-varying variables can be included in FE regression analysis (with one exception: time-constant factors can be included as moderating variables, e.g. depression x sex).

Robust standard errors that cluster errors at the individual level were computed to take serial correlation and heteroscedasticity into consideration. In order to check the robustness of our findings, all analyses were repeated with FE poisson models with cluster-robust standard errors. The P threshold for determination of statistical significance was .05. All statistical analyses were conducted using Stata 13.1 (Stata Corp., College Station, Texas). Moreover, please note that the Stata command for FE regressions included individuals with only one observation in calculating the number of observation since they provide information among others about the constant and the variance components. However, it does not affect the beta-coefficients and the standard errors. Additionally, it is worth mentioning that we did not use weights (calibrated longitudinal weights for the fifth wave were not available to this date) which might affect the estimates, e.g. in terms of efficiency [37]. For further details please see Stuck et al. [38] and Solon et al. [37]. In addition to the regression analysis in older adults (total sample; men and women), we exploratory examined the predictors of FI in very old age (aged 80 and above) as well as in individuals younger than 80 years.

To test the robustness of our findings, sensitivity analysis was conducted. To this end, living situation was removed from the model specification since it might be correlated with family status. Moreover, FE poisson models was used (instead of linear FE regression models).

## Results

### Descriptive results

Our descriptive statistics across waves are depicted in Table 1. We reported descriptive statistics for individuals who reported at least two valid values for ADL 1, ADL 2, IADL 1 and IADL 2.

**Table 1:** Descriptive statistics over time.

Variables	Wave 1 (n = 22,439)	Wave 2 (n = 28,963)	Wave 4 (n = 43,463)	Wave 5 (n = 41,858)
Age^a^: Mean (SD)	63.7 (10.1)	65.2 (10.2)	66.3 (10.1)	67.9 (10.0)
Female: N (%)	12,577 (56.1)	16,218 (56.0)	24,794 (57.1)	23,961 (57.2)
Education (ISCED-97 Coding): N (%)				
Primary education or first stage of basic education	5,980 (28.6)	7,377 (27.2)	8,063 (19.5)	7,220 (18.2)
Lower secondary or second stage of basic education	3,919 (18.8)	4,901 (18.0)	7,994 (19.3)	7,848 (19.7)
(Upper) secondary education	6,052 (29.0)	8,333 (30.7)	14,478 (35.1)	13,768 (34.6)
Post-secondary non-tertiary education	593 (2.8)	936 (3.4)	1,986 (4.8)	2,007 (5.1)
First stage of tertiary education	4,251 (20.3)	5,480 (20.2)	8,409 (20.4)	8,557 (21.5)
Second stage of tertiary education	104 (0.5)	121 (0.5)	357 (0.9)	346 (0.9)
Marital status: N (%)				
Married and living together with spouse	16,220 (72.3)	20,760 (71.9)	29,274 (68.1)	27,432 (66.2)
Registered partnership	345 (1.5)	387 (1.3)	648 (1.5)	620 (1.5)
Married, living separated from spouse	219 (1.0)	313 (1.1)	511 (1.2)	483 (1.2)
Never married	1,129 (5.0)	1,362 (4.7)	2,437 (5.6)	2,341 (5.7)
Divorced	1,392 (6.2)	1,840 (6.4)	3,855 (9.0)	3,825 (9.2)
Widowed	3,131 (14.0)	4,211 (14.6)	6,278 (14.6)	6,706 (16.2)
Living with a spouse/partner in household: N (%)	16,973 (75.8)	21,831 (75.4)	31,275 (72.0)	29,285 (70.0)
Alcohol consumption per week^:^ N (%)				
Daily alcohol consumption	4,819 (21.5)	5,627 (19.6)	7,961 (18.4)	7,273 (17.4)
Less than daily alcohol consumption	17,615 (78.5)	23,128 (80.4)	35,183 (81.6)	34,563 (82.6)
Current smoker: N (%)	4,227 (18.8)	5,370 (18.6)	8,044 (18.6)	7,463 (17.8)
Absence of depression: N (%)	16,949 (75.5)	22,079 (76.2)	31,623 (72.8)	30,853 (73.7)
Cognitive function^b^: Mean (SD)	4.9 (1.8)	5.0 (1.8)	5.2 (1.8)	5.3 (1.9)
Chronic diseases (Count score)^c^: Mean (SD)	3.6 (2.3)	3.4 (2.2)	3.4 (2.2)	3.3 (2.2)
ADL 1: Mean (SD)	0.1 (0.4)	0.1 (0.5)	0.2 (0.5)	0.2 (0.6)
ADL 2: Mean (SD)	0.2 (0.6)	0.2 (0.7)	0.2 (0.7)	0.3 (0.8)
IADL 1: Mean (SD)	0.1 (0.3)	0.1 (0.4)	0.1 (0.4)	0.1 (0.5)
IADL 2: Mean (SD)	0.1 (0.6)	0.2 (0.7)	0.2 (0.7)	0.2 (0.8)

Missing values for metric variables (if occurred)

^a^ 195 missing values in the first wave and 195 missing values in the second wave

^b^ 238 missing values in the first wave, 478 missing values in the second wave, 859 missing values in the fourth wave and 1,225 missing values in the fifth wave

^c^ 5 missing value in the first wave, 208 missing values in the second wave, 319 missing values in the fourth wave and 22 missing values in the fifth wave

SD: Standard deviation

### Regression analysis

Linear FE regressions (Table 2: total sample; Table 3: total sample by gender) showed that FI (all outcome measures) significantly increased with age, less than daily alcohol consumption, cognitive impairment, the onset of depression and increasing chronic conditions in the total sample and in both sexes. Moreover, the transition to ‘not living with a spouse/partner in household’ increased FI in the total sample and in men (all outcome measures) as well as in women (IADL 1, IADL 2). Furthermore, the onset of smoking increased FI in the total sample (all outcome measures), in men (IADL 1, IADL 2) and in women (ADL 1, ADL 2). There was no robust effect of marital status and income on FI.

**Table 2:** Factors affecting functional impairment: Results of linear fixed effects regression analysis (total sample).

	(1)	(2)	(3)	(4)
Variables	ADL 1	ADL 2	IADL 1	IADL 2
Age	0.0118***	0.0164***	0.00786***	0.0183***
	(0.000457)	(0.000639)	(0.000337)	(0.000605)
Without a partner/spouse^a^ (Ref.: Married and living together with spouse/registered partnership)	0.00618	0.0127	0.00677	0.00972
	(0.0163)	(0.0228)	(0.0128)	(0.0229)
Not living with a spouse/partner in household (Ref.: Living with a spouse/partner in household)	0.0214***	0.0287**	0.0199***	0.0446***
	(0.00640)	(0.00906)	(0.00482)	(0.00881)
Household income: above median (Ref.: below median)	0.00820*	0.0121*	0.00155	0.00780+
	(0.00339)	(0.00475)	(0.00251)	(0.00432)
Daily alcohol consumption (Ref.: less than daily alcohol consumption)	-0.0221***	-0.0357***	-0.0174***	-0.0389***
	(0.00475)	(0.00656)	(0.00350)	(0.00609)
Smoking (Ref.: Currently not smoking)	0.00483**	0.00713**	0.00288*	0.00504*
	(0.00170)	(0.00238)	(0.00115)	(0.00210)
Cognitive function	-0.0151***	-0.0246***	-0.0185***	-0.0341***
	(0.00116)	(0.00166)	(0.000997)	(0.00170)
Occurrence of depression (Ref: Absence of depression)	0.0753***	0.105***	0.0356***	0.0870***
	(0.00455)	(0.00646)	(0.00346)	(0.00605)
Chronic diseases (Count score)	0.0296***	0.0398***	0.0121***	0.0288***
	(0.00237)	(0.00344)	(0.00188)	(0.00327)
Constant	-0.667***	-0.902***	-0.416***	-1.005***
	(0.0306)	(0.0428)	(0.0218)	(0.0395)
Observations	179,806	179,806	179,806	179,806
R^2^	0.029	0.030	0.025	0.039
Number of Individuals	103,384	103,384	103,384	103,384

^a^ ‘Without a partner/spouse”: Married, living separated from spouse; never married; divorced; widowed; Cluster-robust standard errors in parentheses

*** p<0.001

** p<0.01

* p<0.05

+ p<0.10

Observations with missing values were dropped (listwise deletion).

**Table 3:** Factors affecting functional impairment: Results of linear fixed effects regression analysis (total sample, by gender).

	(1)	(2)	(3)	(4)	(5)	(6)	(7)	(8)
Variables	ADL 1—Men	ADL 2—Men	IADL 1—Men	IADL 2—Men	ADL 1—Women	ADL 2—Women	IADL 1—Women	IADL 2—Women
Age	0.0110***	0.0150***	0.00733***	0.0169***	0.0123***	0.0173***	0.00822***	0.0193***
	(0.000656)	(0.000913)	(0.000496)	(0.000887)	(0.000631)	(0.000885)	(0.000457)	(0.000824)
Without a partner/spouse^a^ (Ref.: Married and living together with spouse/registered partnership)	-0.0185	-0.0239	-0.0132	-0.00733	0.0215	0.0364	0.0190	0.0236
	(0.0233)	(0.0326)	(0.0191)	(0.0350)	(0.0220)	(0.0309)	(0.0168)	(0.0298)
Not living with a spouse/partner in household (Ref.: Living with a spouse/partner in household)	0.0283**	0.0418**	0.0251**	0.0604***	0.0161+	0.0189	0.0161**	0.0332**
	(0.00947)	(0.0137)	(0.00782)	(0.0144)	(0.00864)	(0.0121)	(0.00612)	(0.0111)
Household income: above median (Ref.: below median)	0.00949+	0.00908	0.000110	0.00540	0.00676	0.0139*	0.00246	0.00910
	(0.00504)	(0.00702)	(0.00377)	(0.00662)	(0.00457)	(0.00644)	(0.00336)	(0.00568)
Daily alcohol consumption (Ref.: less than daily alcohol consumption)	-0.0246***	-0.0425***	-0.0193***	-0.0435***	-0.0190**	-0.0268**	-0.0151**	-0.0331***
	(0.00626)	(0.00869)	(0.00460)	(0.00805)	(0.00725)	(0.00992)	(0.00540)	(0.00927)
Smoking (Ref.: Currently not smoking)	0.00404+	0.00597+	0.00349*	0.00639*	0.00547*	0.00817*	0.00204	0.00328
	(0.00238)	(0.00329)	(0.00173)	(0.00308)	(0.00242)	(0.00342)	(0.00148)	(0.00278)
Cognitive function	-0.0172***	-0.0269***	-0.0179***	-0.0331***	-0.0135***	-0.0227***	-0.0189***	-0.0347***
	(0.00167)	(0.00239)	(0.00150)	(0.00256)	(0.00160)	(0.00229)	(0.00133)	(0.00228)
Occurrence of depression (Ref: Absence of depression)	0.117***	0.163***	0.0550***	0.134***	0.0535***	0.0753***	0.0254***	0.0625***
	(0.00848)	(0.0121)	(0.00647)	(0.0113)	(0.00530)	(0.00751)	(0.00404)	(0.00705)
Chronic diseases (Count score)	0.0334***	0.0427***	0.0159***	0.0313***	0.0265***	0.0375***	0.00917***	0.0269***
	(0.00343)	(0.00496)	(0.00288)	(0.00498)	(0.00326)	(0.00474)	(0.00248)	(0.00431)
Constant	-0.623***	-0.827***	-0.399***	-0.963***	-0.693***	-0.951***	-0.424***	-1.027***
	(0.0447)	(0.0619)	(0.0330)	(0.0596)	(0.0417)	(0.0587)	(0.0289)	(0.0525)
Observations	78,744	78,744	78,744	78,744	101,060	101,060	101,060	101,060
R^2^	0.039	0.040	0.028	0.044	0.024	0.025	0.024	0.037
Number of Individuals	45,789	45,789	45,789	45,789	57,593	57,593	57,593	57,593

^a^ ‘Without a partner/spouse”: Married, living separated from spouse; never married; divorced; widowed; Cluster-robust standard errors in parentheses

*** p<0.001

** p<0.01

* p<0.05

+ p<0.10

Observations with missing values were dropped (listwise deletion).

Since the effect of depression on FI was very high in men (Table 3), we tested whether there are significant differences between men and women (interaction term: gender x depression). Indeed, the interaction term was significant with all outcome measures (in each case with p < .001).

In terms of significance, the findings for individuals aged 80 and above (S1 and S2 Tables) are comparable to the findings for individuals aged <80 years (S3 and S4 Tables). However, while FI did not increase with the onset of smoking in individuals aged 80 and above, FI increased with the onset of smoking in individuals aged <80 years.

### Sensitivity analysis

Since living situation might be correlated with marital status, we removed living situation from our main model in sensitivity analysis (results of alternate models are not shown, but are available upon request from the authors). In this model specification, the effect of marital status was statistically significant in the total sample and in both sexes. In terms of significance, the other predictors remained almost the same. Furthermore, we redid everything with FE poisson models. Findings from these models were very similar in terms of significance levels to those derived from linear FE models.

## Discussion

### Main findings

Longitudinal regressions revealed that FI in older adults increased significantly with age, less than daily alcohol consumption, the occurrence of depression, cognitive impairment, and the number of chronic conditions in the total sample and in both sexes. Moreover, the onset of smoking and living without a spouse/partner in household increased FI in the total sample. The effect of depression on FI was significantly more pronounced in men.

### Previous research

Our findings confirm the hypothesis that the occurrence of depression, cognitive impairment and the number of chronic conditions are causal factors for FI. Therefore, it extends previous knowledge about an association of these variables [6]. The effect of depression on FI may be explained by the fact that the occurrence of depression is associated with less social ties and less physical activities [21, 22]. Moreover, it is reasonable that with increasing cognitive deficits dealing with activities of daily life such as using the telephone becomes increasingly difficult [39, 40]. As for chronic conditions, our findings are difficult to compare with previous studies which found an association between chronic conditions at baseline and subsequent FI [41, 42] due to differences in statistical methods, model specification and the large variety in chronic condition used to estimate comorbidity scores.

Concerning age, our findings correspond to existing literature [8, 9, 15]. The age-effect found in our study is worth underlining because we controlled for sociodemographic factors, depression, cognitive impairment as well as chronic diseases in regression analysis. With regard to living situation, the literature is heterogeneous. Due to the aforementioned tremendous variety in statistical models used, findings are difficult to compare. For instance, our findings and studies that identified the baseline variable ‘living alone’ as a risk factor for subsequent functional decline [43] might be explicated by the fact that living alone reflect other factors increasing the subsequent risk of FI such as multiple falls or decreased physical activity [43].

With less than daily alcohol consumption, FI increased slightly in our study. At first glance, this seems counterintuitive since it is well known that alcohol has numerous negative effects on health. Nevertheless, the effect can be explained by individuals abstaining because of poor health [44]. This is supported by an additional model (not reported in sensitivity analyses) where we did not control for cognitive function and, consequently, the slight alcohol effect considerably increased. Thus, alcohol consumption is positively correlated (r = .12) with cognitive function (the more alcohol consumption the more cognitive function). Moreover, the effect of smoking in older adults is in accordance with previous studies [45, 46]. The non-significant effect of smoking in individuals in very old age might be explained by the limited statistical power (since most individuals in very old age did not quit or start smoking).

### Strengths and limitations

This is the first study investigating the longitudinal predictors of FI in older adults in Europe in the long run (2004–2013). Moreover, it should be highlighted that by using FE regressions unobserved heterogeneity can be taken into account, providing consistent estimates under the assumption of strict exogeneity. Another strength is that our outcome variable, FI, was assessed using validated instruments to quantify impairments in basic as well as instrumental activities of daily living.

However, the assessment of FI was self-reported and thus might be slightly overestimated [47]. Moreover, even though validated measures was used in all countries, we cannot rule out the possibility that cross-national differences exist in understanding the items. Additionally, reverse causality between depression and functional decline might exist [48]. Theoretically, this can be solved by using panel instrumental variable regressions [34]. Empirically, the problem of weak instruments was present in our case. Furthermore, our estimates might be biased downwards due to attrition. Attrition bias was already shown in SHARE data set by Palgi et al. [49], nevertheless the size of the differences between individuals who only participated in wave 1 and individuals who participated at least twice was negligible. Generally, the loss of observations due to the standard approach in microeconometrics (listwise deletion) leads to less inference and precise estimation. Moreover, it might lead to sample-selection bias [50].

## Conclusions

In sum, our findings emphasize the importance of age, the occurrence of depression, cognitive impairments, smoking and chronic conditions for impairments in basic and instrumental activities of daily living. While some of these factors in older adults are inevitable, some may be not, especially depression and smoking. Consequently, in order to prevent or postpone FI in older adults, generating interventions to prevent depression or stop smoking might be productive strategies.

## Supporting Information

S1 Table Factors affecting functional impairment: Results of linear fixed effects regression analysis (age ≥80 years) ^a^ ‘Without a partner/spouse”: Married, living separated from spouse; never married; divorced; widowed; Cluster-robust standard errors in parentheses; *** p<0.001, ** p<0.01, * p<0.05, + p<0.10; Observations with missing values were dropped (listwise deletion). (DOCX)

S2 Table Factors affecting functional impairment: Results of linear fixed effects regression analysis (age ≥80 years, by gender) ^a^ ‘Without a partner/spouse”: Married, living separated from spouse; never married; divorced; widowed; Cluster-robust standard errors in parentheses; *** p<0.001, ** p<0.01, * p<0.05, + p<0.10; Observations with missing values were dropped (listwise deletion). (DOCX)

S3 Table Factors affecting functional impairment: Results of linear fixed effects regression analysis (age <80 years) ^a^ ‘Without a partner/spouse”: Married, living separated from spouse; never married; divorced; widowed; Cluster-robust standard errors in parentheses; *** p<0.001, ** p<0.01, * p<0.05, + p<0.10; Observations with missing values were dropped (listwise deletion). (DOCX)

S4 Table Factors affecting functional impairment: Results of linear fixed effects regression analysis (age <80 years, by gender) ^a^ ‘Without a partner/spouse”: Married, living separated from spouse; never married; divorced; widowed; Cluster-robust standard errors in parentheses; *** p<0.001, ** p<0.01, * p<0.05, + p<0.10; Observations with missing values were dropped (listwise deletion). (DOCX)
