# Supplementary material for: Longitudinal Predictors of Functional Impairment in Older Adults in Europe – Evidence from the Survey of Health, Ageing and Retirement in Europe
Source: PLoS One. 2016 Jan 19;11(1):e0146967. doi: 10.1371/journal.pone.0146967 (PMC4718586; doi:10.1371/journal.pone.0146967)
Supplement: S1 Table — (DOCX) [file pone.0146967.s001.docx]

**S1 Table. Factors affecting functional impairment: Results of linear fixed effects regression analysis (age ≥80 years)**

|  | (1) | (2) | (3) | (4) |
| --- | --- | --- | --- | --- |
| Variables | ADL 1 | ADL 2 | IADL 1 | IADL 2 |
|  |  |  |  |  |
| Age | 0.0594*** | 0.0859*** | 0.0526*** | 0.122*** |
|  | (0.00355) | (0.00515) | (0.00323) | (0.00556) |
| Without a partner/spouse^a^ (Ref.: Married and living together with spouse/registered partnership) | 0.0406 | 0.0527 | 0.0846 | 0.0698 |
|  | (0.0761) | (0.112) | (0.0701) | (0.123) |
| Not living with a spouse/partner in household (Ref.: Living with a spouse/partner in household) | 0.00101 | 0.0127 | 0.000937 | 0.0217 |
|  | (0.0329) | (0.0485) | (0.0298) | (0.0519) |
| Household income: above median (Ref.: below median) | 0.0231 | 0.0267 | -0.0142 | -0.0185 |
|  | (0.0238) | (0.0342) | (0.0210) | (0.0350) |
| Daily alcohol consumption (Ref.: less than daily alcohol consumption) | -0.0598* | -0.120** | -0.0706** | -0.130** |
|  | (0.0300) | (0.0436) | (0.0270) | (0.0459) |
| Smoking (Ref.: Currently not smoking) | 0.00724 | 0.0121 | 0.0214+ | 0.0430* |
|  | (0.0136) | (0.0197) | (0.0112) | (0.0183) |
| Cognitive function | -0.0342*** | -0.0582*** | -0.0590*** | -0.0977*** |
|  | (0.00674) | (0.00966) | (0.00621) | (0.0104) |
| Occurrence of depression (Ref: Absence of depression) | 0.139*** | 0.186*** | 0.0718*** | 0.176*** |
|  | (0.0227) | (0.0331) | (0.0203) | (0.0340) |
| Chronic diseases (Count score) | 0.0487*** | 0.0676*** | 0.0442*** | 0.0951*** |
|  | (0.00901) | (0.0136) | (0.00853) | (0.0141) |
| Constant | -4.635*** | -6.695*** | -4.158*** | -9.749*** |
|  | (0.303) | (0.439) | (0.271) | (0.468) |
|  |  |  |  |  |
| Observations | 18,423 | 18,423 | 18,423 | 18,423 |
| R² | 0.096 | 0.101 | 0.112 | 0.170 |
| Number of Individuals | 12,721 | 12,721 | 12,721 | 12,721 |

^a^ ‘Without a partner/spouse”: Married, living separated from spouse; never married; divorced; widowed; Cluster-robust standard errors in parentheses; *** p<0.001, ** p<0.01, * p<0.05, + p<0.10; Observations with missing values were dropped (listwise deletion).
