# Supplementary material for: Longitudinal Predictors of Functional Impairment in Older Adults in Europe – Evidence from the Survey of Health, Ageing and Retirement in Europe
Source: PLoS One. 2016 Jan 19;11(1):e0146967. doi: 10.1371/journal.pone.0146967 (PMC4718586; doi:10.1371/journal.pone.0146967)
Supplement: S2 Table — (DOCX) [file pone.0146967.s002.docx]

**S2 Table. Factors affecting functional impairment: Results of linear fixed effects regression analysis (age ≥80 years, by gender)**

|  | (1) | (2) | (3) | (4) | (5) | (6) | (7) | (8) |
| --- | --- | --- | --- | --- | --- | --- | --- | --- |
| Variables | ADL 1 - Men | ADL 2 - Men | IADL 1 - Men | IADL 2 - Men | ADL 1 - Women | ADL 2 - Women | IADL 1 - Women | IADL 2 - Women |
|  |  |  |  |  |  |  |  |  |
| Age | 0.0504*** | 0.0720*** | 0.0470*** | 0.110*** | 0.0646*** | 0.0937*** | 0.0558*** | 0.129*** |
|  | (0.00524) | (0.00764) | (0.00504) | (0.00884) | (0.00472) | (0.00682) | (0.00416) | (0.00712) |
| Without a partner/spouse^a^ (Ref.: Married and living together with spouse/registered partnership) | 0.00955 | 0.0450 | 0.0979 | 0.0302 | 0.0482 | 0.0211 | 0.0510 | 0.0619 |
|  | (0.108) | (0.150) | (0.0951) | (0.166) | (0.105) | (0.162) | (0.100) | (0.176) |
| Not living with a spouse/partner in household (Ref.: Living with a spouse/partner in household) | 0.000864 | -0.000718 | -0.0228 | 0.0180 | 0.00993 | 0.0417 | 0.0316 | 0.0419 |
|  | (0.0475) | (0.0652) | (0.0404) | (0.0702) | (0.0446) | (0.0700) | (0.0429) | (0.0752) |
| Household income: above median (Ref.: below median) | 0.0139 | -0.0112 | -0.0352 | -0.0814+ | 0.0343 | 0.0632 | 0.00622 | 0.0383 |
|  | (0.0312) | (0.0460) | (0.0272) | (0.0468) | (0.0350) | (0.0495) | (0.0313) | (0.0509) |
| Daily alcohol consumption (Ref.: less than daily alcohol consumption) | -0.0284 | -0.100+ | -0.0598+ | -0.132* | -0.106* | -0.155* | -0.0883* | -0.142+ |
|  | (0.0378) | (0.0542) | (0.0332) | (0.0579) | (0.0475) | (0.0695) | (0.0439) | (0.0724) |
| Smoking (Ref.: Currently not smoking) | -0.00978 | -0.00944 | 0.0107 | 0.0212 | 0.0269 | 0.0373 | 0.0340* | 0.0681** |
|  | (0.0199) | (0.0300) | (0.0173) | (0.0272) | (0.0182) | (0.0252) | (0.0142) | (0.0243) |
| Cognitive function | -0.0368*** | -0.0494** | -0.0503*** | -0.0804*** | -0.0317*** | -0.0620*** | -0.0634*** | -0.107*** |
|  | (0.0108) | (0.0154) | (0.0102) | (0.0173) | (0.00861) | (0.0124) | (0.00781) | (0.0130) |
| Occurrence of depression (Ref: Absence of depression) | 0.231*** | 0.322*** | 0.114** | 0.325*** | 0.0899** | 0.114** | 0.0503* | 0.0967* |
|  | (0.0384) | (0.0558) | (0.0356) | (0.0599) | (0.0281) | (0.0411) | (0.0245) | (0.0408) |
| Chronic diseases (Count score) | 0.0684*** | 0.0988*** | 0.0660*** | 0.127*** | 0.0376*** | 0.0506** | 0.0321** | 0.0781*** |
|  | (0.0152) | (0.0235) | (0.0147) | (0.0242) | (0.0111) | (0.0163) | (0.0103) | (0.0171) |
| Constant | -3.865*** | -5.556*** | -3.675*** | -8.739*** | -5.139*** | -7.426*** | -4.500*** | -10.39*** |
|  | (0.438) | (0.637) | (0.424) | (0.741) | (0.412) | (0.594) | (0.352) | (0.605) |
|  |  |  |  |  |  |  |  |  |
| Observations | 7,495 | 7,495 | 7,495 | 7,495 | 10,928 | 10,928 | 10,928 | 10,928 |
| R² | 0.113 | 0.116 | 0.115 | 0.177 | 0.093 | 0.099 | 0.114 | 0.172 |
| Number of Individuals | 5,268 | 5,268 | 5,268 | 5,268 | 7,453 | 7,453 | 7,453 | 7,453 |

^a^ ‘Without a partner/spouse”: Married, living separated from spouse; never married; divorced; widowed; Cluster-robust standard errors in parentheses; *** p<0.001, ** p<0.01, * p<0.05, + p<0.10; Observations with missing values were dropped (listwise deletion).
