# Supplementary material for: Longitudinal Predictors of Functional Impairment in Older Adults in Europe – Evidence from the Survey of Health, Ageing and Retirement in Europe
Source: PLoS One. 2016 Jan 19;11(1):e0146967. doi: 10.1371/journal.pone.0146967 (PMC4718586; doi:10.1371/journal.pone.0146967)
Supplement: S3 Table — (DOCX) [file pone.0146967.s003.docx]

**S3 Table. Factors affecting functional impairment: Results of linear fixed effects regression analysis (age <80 years)**

|  | (1) | (2) | (3) | (4) |
| --- | --- | --- | --- | --- |
| Variables | ADL 1 - All | ADL 2 - All | IADL 1 - All | IADL 2 - All |
|  |  |  |  |  |
| Age | 0.00679*** | 0.00929*** | 0.00351*** | 0.00836*** |
|  | (0.000420) | (0.000580) | (0.000276) | (0.000491) |
| Without a partner/spouse^a^ (Ref.: Married and living together with spouse/registered partnership) | 0.00340 | 0.00178 | -0.0101 | -0.00895 |
|  | (0.0150) | (0.0207) | (0.0106) | (0.0193) |
| Not living with a spouse/partner in household (Ref.: Living with a spouse/partner in household) | 0.00645 | 0.00556 | 0.00957* | 0.0172* |
|  | (0.00588) | (0.00824) | (0.00377) | (0.00711) |
| Household income: above median (Ref.: below median) | 0.00325 | 0.00546 | -0.000367 | 0.00245 |
|  | (0.00309) | (0.00426) | (0.00209) | (0.00361) |
| Daily alcohol consumption (Ref.: less than daily alcohol consumption) | -0.0124** | -0.0188** | -0.00756** | -0.0177*** |
|  | (0.00439) | (0.00587) | (0.00285) | (0.00500) |
| Smoking (Ref.: Currently not smoking) | 0.00529** | 0.00722** | 0.00266* | 0.00558** |
|  | (0.00165) | (0.00227) | (0.00105) | (0.00197) |
| Cognitive function | -0.00875*** | -0.0151*** | -0.00997*** | -0.0189*** |
|  | (0.00104) | (0.00149) | (0.000825) | (0.00142) |
| Occurrence of depression (Ref: Absence of depression) | 0.0614*** | 0.0870*** | 0.0291*** | 0.0693*** |
|  | (0.00426) | (0.00594) | (0.00296) | (0.00518) |
| Chronic diseases (Count score) | 0.0267*** | 0.0348*** | 0.00761*** | 0.0197*** |
|  | (0.00230) | (0.00327) | (0.00161) | (0.00284) |
| Constant | -0.356*** | -0.462*** | -0.164*** | -0.412*** |
|  | (0.0275) | (0.0380) | (0.0175) | (0.0316) |
|  |  |  |  |  |
| Observations | 161,383 | 161,383 | 161,383 | 161,383 |
| R² | 0.018 | 0.018 | 0.011 | 0.019 |
| Number of Individuals | 94,358 | 94,358 | 94,358 | 94,358 |

^a^ ‘Without a partner/spouse”: Married, living separated from spouse; never married; divorced; widowed; Cluster-robust standard errors in parentheses; *** p<0.001, ** p<0.01, * p<0.05, + p<0.10; Observations with missing values were dropped (listwise deletion).
