# Supplementary material for: Longitudinal Predictors of Functional Impairment in Older Adults in Europe – Evidence from the Survey of Health, Ageing and Retirement in Europe
Source: PLoS One. 2016 Jan 19;11(1):e0146967. doi: 10.1371/journal.pone.0146967 (PMC4718586; doi:10.1371/journal.pone.0146967)
Supplement: S4 Table — (DOCX) [file pone.0146967.s004.docx]

|  | (1) | (2) | (3) | (4) | (5) | (6) | (7) | (8) |
| --- | --- | --- | --- | --- | --- | --- | --- | --- |
| Variables | ADL 1 - Men | ADL 2 - Men | IADL 1 - Men | IADL 2 - Men | ADL 1 - Women | ADL 2 - Women | IADL 1 - Women | IADL 2 - Women |
|  |  |  |  |  |  |  |  |  |
| Age | 0.00677*** | 0.00917*** | 0.00392*** | 0.00894*** | 0.00674*** | 0.00932*** | 0.00315*** | 0.00786*** |
|  | (0.000614) | (0.000836) | (0.000427) | (0.000751) | (0.000573) | (0.000799) | (0.000360) | (0.000647) |
| Without a partner/spouse^a^ (Ref.: Married and living together with spouse/registered partnership) | -0.0226 | -0.0427 | -0.0289* | -0.0229 | 0.0201 | 0.0316 | 0.00119 | 0.00139 |
|  | (0.0201) | (0.0270) | (0.0147) | (0.0281) | (0.0208) | (0.0287) | (0.0144) | (0.0255) |
| Not living with a spouse/partner in household (Ref.: Living with a spouse/partner in household) | 0.0126 | 0.0198 | 0.0120* | 0.0236* | 0.00181 | -0.00477 | 0.00792 | 0.0131 |
|  | (0.00849) | (0.0123) | (0.00602) | (0.0117) | (0.00807) | (0.0111) | (0.00484) | (0.00896) |
| Household income: above median (Ref.: below median) | 0.00410 | 0.00376 | -0.000681 | 0.00360 | 0.00228 | 0.00634 | -0.000258 | 0.00124 |
|  | (0.00470) | (0.00636) | (0.00338) | (0.00590) | (0.00410) | (0.00574) | (0.00261) | (0.00446) |
| Daily alcohol consumption (Ref.: less than daily alcohol consumption) | -0.0169** | -0.0269** | -0.00952* | -0.0194** | -0.00607 | -0.00747 | -0.00468 | -0.0150* |
|  | (0.00601) | (0.00819) | (0.00395) | (0.00702) | (0.00627) | (0.00807) | (0.00398) | (0.00682) |
| Smoking (Ref.: Currently not smoking) | 0.00455* | 0.00588+ | 0.00340* | 0.00720* | 0.00592* | 0.00850* | 0.00169 | 0.00358 |
|  | (0.00227) | (0.00305) | (0.00155) | (0.00286) | (0.00239) | (0.00338) | (0.00138) | (0.00265) |
| Cognitive function | -0.0115*** | -0.0191*** | -0.0116*** | -0.0215*** | -0.00653*** | -0.0118*** | -0.00869*** | -0.0169*** |
|  | (0.00149) | (0.00212) | (0.00127) | (0.00216) | (0.00144) | (0.00207) | (0.00108) | (0.00188) |
| Occurrence of depression (Ref: Absence of depression) | 0.0961*** | 0.134*** | 0.0431*** | 0.101*** | 0.0434*** | 0.0627*** | 0.0217*** | 0.0527*** |
|  | (0.00812) | (0.0114) | (0.00570) | (0.00995) | (0.00488) | (0.00680) | (0.00337) | (0.00591) |
| Chronic diseases (Count score) | 0.0270*** | 0.0331*** | 0.00821*** | 0.0178*** | 0.0263*** | 0.0362*** | 0.00704*** | 0.0211*** |
|  | (0.00326) | (0.00456) | (0.00246) | (0.00428) | (0.00321) | (0.00462) | (0.00213) | (0.00380) |
| Constant | -0.348*** | -0.448*** | -0.186*** | -0.454*** | -0.359*** | -0.470*** | -0.144*** | -0.373*** |
|  | (0.0407) | (0.0551) | (0.0272) | (0.0492) | (0.0371) | (0.0521) | (0.0227) | (0.0412) |
|  |  |  |  |  |  |  |  |  |
| Observations | 71,249 | 71,249 | 71,249 | 71,249 | 90,132 | 90,132 | 90,132 | 90,132 |
| R² | 0.026 | 0.026 | 0.015 | 0.024 | 0.014 | 0.015 | 0.009 | 0.016 |
| Number of Individuals | 42,124 | 42,124 | 42,124 | 42,124 | 52,232 | 52,232 | 52,232 | 52,232 |

**S4 Table. Factors affecting functional impairment: Results of linear fixed effects regression analysis (age <80 years, by gender)**

^a^ ‘Without a partner/spouse”: Married, living separated from spouse; never married; divorced; widowed; Cluster-robust standard errors in parentheses; *** p<0.001, ** p<0.01, * p<0.05, + p<0.10; Observations with missing values were dropped (listwise deletion).
